# Biochemical Characterization of Oyster and Clam Galectins: Selective Recognition of Carbohydrate Ligands on Host Hemocytes and *Perkinsus* Parasites

**DOI:** 10.3389/fchem.2020.00098

**Published:** 2020-02-25

**Authors:** Gerardo R. Vasta, Chiguang Feng, Satoshi Tasumi, Kelsey Abernathy, Mario A. Bianchet, Iain B. H. Wilson, Katharina Paschinger, Lai-Xi Wang, Muddasar Iqbal, Anita Ghosh, Mohammed N. Amin, Brina Smith, Sean Brown, Aren Vista

**Affiliations:** ^1^Department of Microbiology and Immunology, University of Maryland School of Medicine, Institute of Marine and Environmental Technology, Baltimore, MD, United States; ^2^Departments of Neurology, and Biophysics and Biophysical Chemistry, The Johns Hopkins University School of Medicine, Baltimore, MD, United States; ^3^Department für Chemie, Universität für Bodenkultur, Vienna, Austria; ^4^Department of Chemistry and Biochemistry, University of Maryland, College Park, MD, United States; ^5^Coppin State University, Baltimore, MD, United States; ^6^University of Maryland Baltimore County, Baltimore, MD, United States

**Keywords:** galectin, biochemical characterization, carbohydrate recognition, bivalve hemocyte, perkinsus parasites

## Abstract

Both vertebrates and invertebrates display active innate immune mechanisms for defense against microbial infection, including diversified repertoires of soluble and cell-associated lectins that can effect recognition and binding to potential pathogens, and trigger downstream effector pathways that clear them from the host internal milieu. Galectins are widely distributed and highly conserved lectins that have key regulatory effects on both innate and adaptive immune responses. In addition, galectins can bind to exogenous (“non-self”) carbohydrates on the surface of bacteria, enveloped viruses, parasites, and fungi, and function as recognition receptors and effector factors in innate immunity. Like most invertebrates, eastern oysters (*Crassostrea virginica*) and softshell clams (*Mya arenaria*) can effectively respond to most immune challenges through soluble and hemocyte-associated lectins. The protozoan parasite *Perkinsus marinus*, however, can infect eastern oysters and cause “Dermo” disease, which is highly detrimental to both natural and farmed oyster populations. The sympatric *Perkinsus chesapeaki*, initially isolated from infected *M. arenaria* clams, can also be present in oysters, and there is little evidence of pathogenicity in either clams or oysters. In this review, we discuss selected observations from our studies on the mechanisms of *Perkinsus* recognition that are mediated by galectin-carbohydrate interactions. We identified in the oyster two galectins that we designated CvGal1 and CvGal2, which strongly recognize *P. marinus* trophozoites. In the clam we also identified galectin sequences, and focused on one (that we named MaGal1) that also recognizes *Perkinsus* species. Here we describe the biochemical characterization of CvGal1, CvGal2, and MaGal1 with focus on the detailed study of the carbohydrate specificity, and the glycosylated moieties on the surfaces of the oyster hemocytes and the two *Perkinsus* species (*P. marinus* and *P. chesapeaki*). Our goal is to gain further understanding of the biochemical basis for the interactions that lead to recognition and opsonization of the *Perkinsus* trophozoites by the bivalve hemocytes. These basic studies on the biology of host-parasite interactions may contribute to the development of novel intervention strategies for parasitic diseases of biomedical interest.

## Introduction

In both invertebrates and vertebrates, the immediate recognition of surface moieties on the surface of potential pathogens and parasites or their soluble extracellular products is a critical step for a successful innate immune response (Janeway and Medzhitov, 2002). Among these, bacterial lipopolysaccharides and exopolysaccharides, viral envelope glycoproteins, and surface carbohydrate structures from eukaryotic parasites encode complex information that is “decoded” by the host's soluble or membrane-associated carbohydrate-binding proteins (lectins) (Laine, 1997). Upon recognition and binding to exogenous carbohydrate moieties, lectins can activate signaling pathways and initiate downstream events that include agglutination, immobilization, and opsonization, and activation of effector pathways, such as prophenoloxidase and complement, that can promote killing and clearance of the potential pathogen or parasite (Vasta and Ahmed, 2008). Therefore, lectins are key recognition and effector factors of innate immune responses (Vasta et al., 2007). Most lectins are oligomeric associations of peptide subunits that can be covalently or non-covalently bound, and are characterized by the presence of one or more carbohydrate recognition domains (CRDs) (Vasta et al., 2007; Vasta and Ahmed, 2008)^1^. Initially based on canonical amino acid sequence motifs in their CRD, and most recently on their structural fold, lectins are currently classified in distinct major families, such as galectins (formerly S-type lectins), C-, P-, X-, and I-types, and others (Vasta et al., 2007; Vasta and Ahmed, 2008)^1^. In addition to a unique sequence motif in their CRD and their structural fold, galectins are characterized by their binding preference for β-galactosides, wide taxonomic distribution (Leffler et al., 2004; Vasta and Ahmed, 2008), and functional diversification (Leffler et al., 2004; Vasta, 2009; Rabinovich and Croci, 2012; Vasta et al., 2012). By binding to endogenous (“self”) glycans, galectins mediate developmental processes (Leffler et al., 2004; Vasta and Ahmed, 2008), and regulate immune responses (Rabinovich and Croci, 2012). Galectins also bind exogenous (“non-self”) glycans on the surface of viruses, bacteria, and parasites, and participate in innate immunity (Vasta, 2009; Vasta et al., 2012).

**Figure 1:**
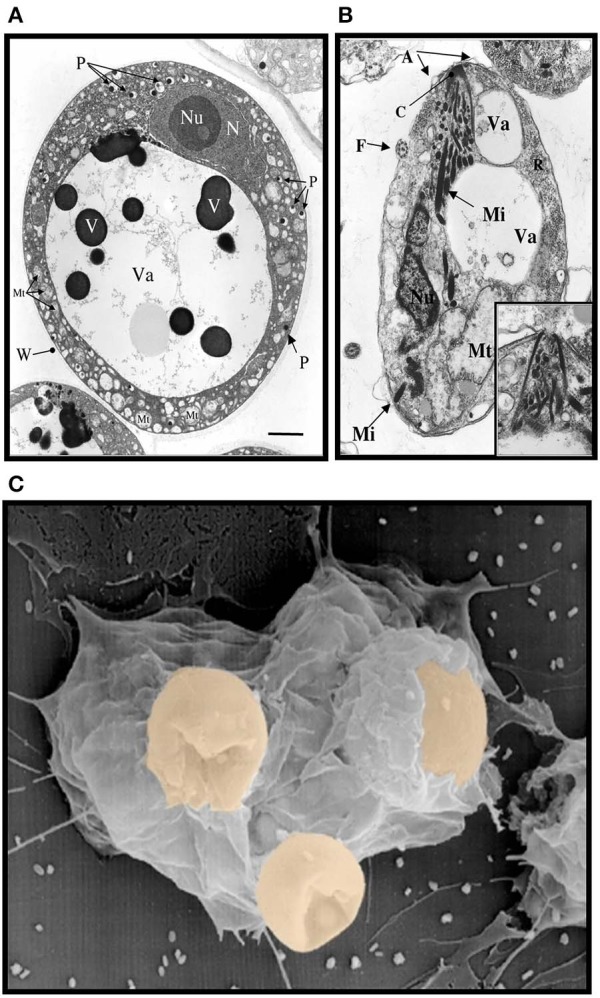
Electron micrographs of *Perkinsus* sp. **(A)** Mature trophozoites of *Perkinsus* sp. isolated from the Baltic clam, *Macoma balthica*. Scale bar = 2 μm. A, cortical alveoli expansion; Mt, mitochondria; N, nucleus; Nu, nucleolus; P, vacuoplast precursors; Va, vacuole; W, wall; Mi, rectilinear micronemes; F, flagellum; C; conoid. Scale bar = 1 μm. **(B)** Longitudinal section of mature zoospores of *Perkinsus* sp. isolated from *Macoma balthica*. Insert shows detail of anterior end of another zoospore. **(C)** Phagocytosis of *Perkinsus marinus* by eastern oyster (*Crassostrea virginica*) hemocytes in a scanning electron micrograph [Adapted from Harvell et al. (1999) with permission from the American Association for the Advancement of Science].

Like most invertebrates, eastern oysters (*Crassostrea virginica*) and softshell clams (*Mya arenaria*) lack the typical adaptive immune responses of vertebrates mediated by immunoglobulins, B and T cells, and rely upon innate immune responses for defense against infection (Janeway and Medzhitov, 2002). This comprises both cellular and humoral defense responses initiated by multiple cell-associated and soluble receptors, including galectins that can recognize the infectious challenge and lead to effector functions, such as opsonization and phagocytosis by hemolymph cells (hemocytes). Thus, invertebrates, including oysters and softshell clams can effectively respond to most immune challenges through soluble and hemocyte-associated recognition and effector factors (Vasta et al., 1982, 1984, 2004a; Kennedy et al., 1996; Dame et al., 2002). The eukaryotic parasite *Perkinsus marinus*, however, successfully infects the oyster and causes “Dermo” disease along the east and Gulf coasts of the USA, resulting in mass mortalities with serious consequences for both natural and farmed shellfisheries, and the water quality of coastal environments (Andrews, 1996; Kennedy et al., 1996; Perkins, 1996; Harvell et al., 1999; Dame et al., 2002; Caceres-Martinez et al., 2012). Transmission of the parasite from infected oysters is thought to occur through the release of *P. marinus* trophozoites, which are filtered by the healthy oysters together with the phytoplankton (Figure 1A). Trophozoites released into the water column can mature into hypnospores that release numerous flagellated zoospores, but their potential infective capacity is not fully understood (Figure 1B). Once in contact with the mucosal surfaces, trophozoites are phagocytosed by hemocytes (Figure 1C), survive intracellular killing, and proliferate, causing systemic infection and death of the oyster (Chu, 1996; Bushek et al., 2002; Ford et al., 2002). The sympatric *Perkinsus chesapeaki*, initially isolated from infected *M. arenaria* clams, can also be present in oysters, but there is little evidence of pathogenicity for either bivalve species. The detailed mechanisms of parasite recognition and entry, and the determinants of host preference and pathogenicity of *Perkinsus* species remain to be fully understood (Reece et al., 2008).

In this review, we discuss selected observations from our studies aimed at gaining further insight into the molecular basis of recognition and opsonization of *Perkinsus* trophozoites by the bivalve hemocytes. During our initial studies on the oyster and the clam, we examined the possibility that recognition of *Perkinsus* parasites by their phagocytic hemocytes could be mediated by protein-carbohydrate interactions. Our results revealed complex lectin repertoires in both bivalve species, among which we identified novel galectins. We then used biochemical, molecular, glycomic, and structural approaches to address the carbohydrate specificity of the oyster and clam galectins, and the identification of glycosylated moieties on the surfaces of the hemocytes and the *Perkinsus* parasites that may be responsible for the host-parasite interactions.

## Identification and Recombinant Expression of Oyster and Clam Galectins: Interactions With Sympatric *Perkinsus* Species

Mining public genomic and EST databases from the oyster *C. virginica* revealed the presence of multiple galactosyl-binding lectins. Their sequences indicated that these belong either to the C-type lectin or galectin families. Based on their domain organization, galectins from vertebrate species are currently classified as “proto”, “chimera,” and “tandem-repeat” types, each endowed with unique molecular structure, biochemical properties, and taxonomic distribution (Hirabayashi and Kasai, 1993). Proto type galectins contain one CRD per subunit, and are usually homodimers of non-covalently-linked subunits. Chimera type galectins comprise a C-terminal CRD and an proline-rich N-terminal domain that participates in subunit oligomerization. In the tandem-repeat galectins, two CRDs are joined by a linker peptide. Surprisingly, the oyster sequence identified as a galectin, revealed the presence of four tandemly arrayed CRDs, which represents a novel feature for a member of the galectin family, and poses interesting questions about its structural and functional aspects. The oyster galectin, which we designated as CvGal1 (*C. virginica* galectin 1), contained most residues responsible for recognition of galactosyl moieties in the four CRDs and therefore, was considered as a potential receptor for *P. marinus* trophozoites (Tasumi and Vasta, 2007; Feng et al., 2013). To test this possibility, we examined the presence of galectin transcripts in oyster hemocytes and selected tissues (gills, gut, muscle, and mantle) by RT-PCR (Tasumi and Vasta, 2007). The results clearly indicated that both CvGal1 is expressed in all tissues tested and based on the similar intensity of the amplicons, it seemed likely that the signals observed in the tissues tested originated from the hemocytes that infiltrate these tissues. Further, it is noteworthy that hemocytes, gills, gut, and mantle, which are cells and tissues that are in direct contact with the external environment, have all been proposed as portals for *P. marinus* infection (Chu, 1996; Bushek et al., 2002; Ford et al., 2002; Reece et al., 2008).

**Figure 2:**
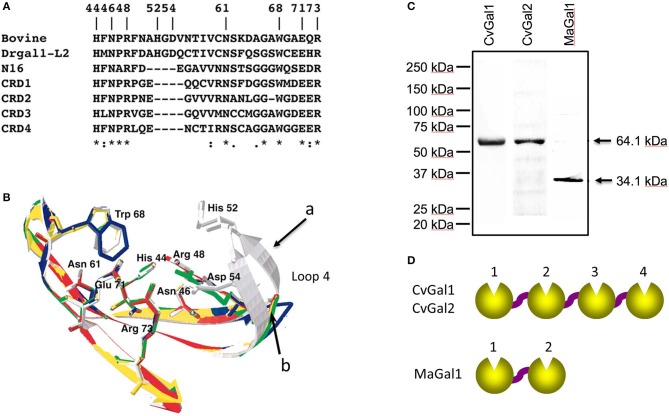
Galectins in bivalves. **(A)** Alignment of bovine galectin−1, zebrafish Drgal1−L2, *C. elegans* LEC−6 (formerly N16), and CRD1 to −4 of CvGal1. **(B)** Homology modeling of CvGal1 CRDs. Bovine galectin−1, CRD−1,−2,−3, and−4 are shown in white, blue, yellow, red, and green, respectively. Numbering of amino acid residues is based on bovine galectin−1. Loop 4 of the CvGal1 CRDs (arrow b) is shorter than the loop 4 of BaGal1 (arrow a). **(C)** Coomassie stain of recombinant CvGal1, CvGal2, and MaGal1 containing either four or two CRDs. Arrows indicate the expected molecular weight of the proteins. **(D)** Schematic illustration of four CRDs of CvGal1 and CvGal2 and two CRDs of MaGal1 [Adapted from Tasumi and Vasta (2007) with permission from the American Association of Immunologists].

To gain further understanding of the oyster's galectin repertoire and the recognition and effector function(s) of its members, we screened the oyster cDNA library to search for proteins that may display the galectin canonical sequence motif. This search identified a second novel galectin which we named CvGal2 (*C. virginica* galectin 2), that was also expressed mostly in the oyster hemocytes, and displayed four tandemly arrayed similar but yet distinct CRDs (Feng et al., 2015). It is noteworthy that the preliminary sequence alignment of CvGal1 CRDs with those of bovine, zebrafish and *C. elegans* galectins showed that two amino acid residues (His^52^ and Asp^54^, based on the mammalian numbering), that interact with the nitrogen of the *N*-acetyl group of the sugar, are missing in all four CvGal1 CRDs (Figure 2A). The oyster galectin CvGal2 also displays short forms of loop 4 in its four CRDs (Feng et al., 2015). The missing tetrapeptide includes His^52^ and Asp^54^ (based on the mammalian numbering; Liao et al., 1994; Figure 2B), which interact with the *N*-acetyllactosamine ligand. Although all four CRDs of the oyster galectins CvGal1 and CvGal2 have very similar primary structure, as in the case of the *C. elegans* galectin LEC-6 (formerly, N16; Tasumi and Vasta, 2007; Feng et al., 2015), the shorter loop 4 and the lack of His^52^ and Asp^54^ confers a different carbohydrate specificity (Liao et al., 1994; Ahmed et al., 2002; Tasumi and Vasta, 2007; Feng et al., 2013, 2015).

Subsequently, we carried out an RNAseq-based transcriptomic analysis of tissues from the softshell clam *M. arenaria* (a bivalve species sympatric with the eastern oyster *C. virginica* in Chesapeake Bay), and identified a galectin-encoding sequence with two tandemly arrayed CRDs, which we designated MaGal1. The two CRDs of MaGal1 also display a short loop 4, and like the oyster galectins CvGal1 and CvGal2, the MaGal1 transcript is mostly expressed in hemocytes (Tasumi and Vasta, 2007; Feng et al., 2013, 2015; Vasta et al., 2015).

**Figure 3:**
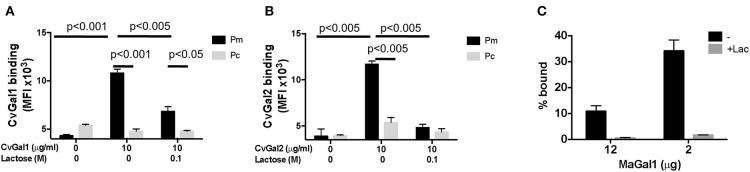
Selective binding of CvGals and MaGal to Perkinsus parasites. **(A,B)** *Perkinsus marinus* (Pm) or *P. chesapeaki* (Pc) were incubated with recombinant CvGal1 **(A)** and CvGal2 **(B)** with or without 0.1 M lactose. The galectin binding was detected with galectin-specific antibodies followed by Fitc-conjugated anti-rabbit secondary antibody incubated, and analyzed in C6 cytometer. **(C)** *P. chesapeaki* was incubated with purified MaGal1 (12 or 2 μg) with (+Lac) or without (–) 0.1 M lactose and the bound galectin was pelleted along with the parasites by centrifugation. The bound galectin was eluted and run in SDS-PAGE along with the unbound galectin remaining in supernatant, and subjected to Coomassie staining. The intensities of both fractions from each samples were quantified (NIH Image J) and % bound was calculated as bound/(bound + unbound) [Adapted from Feng et al. (2015) with permission from the American Chemical Society].

The identification of galectins in oyster and clam hemocytes, the phagocytic cells that are the primary defense mechanism in these filter-feeding bivalves, supported our hypothesis that they may function as *Perkinsus* receptors, and we proceeded to develop tools to enable structural and functional studies on these proteins, with focus on their potential role(s) in parasite recognition and host entry. For this, we started by elucidating the full cDNA and gene sequences, and expressed the recombinant proteins (Tasumi and Vasta, 2007; Feng et al., 2013, 2015). The electrophoretic mobilities of the recombinant proteins rCvGal1, rCvGal2, and rMaGal1 are shown in Figure 2C. The relative electrophoretic mobility of rCvGal1 and rCvGal2, and rMaGal1 confirmed the molecular mass and CRD organization expected from the transcripts' sequences, illustrated in Figure 2D. The recognition properties of rCvGal1, rCvGal2, and rMaGal1 for the two sympatric *Perkinsus* species of interest, *P. marinus* and *P. chesapeaki*, are shown in Figure 3. Most interesting was the observation that while CvGal1 and CvGal2 strongly recognize *P. marinus*, the recognition of *P. chesapeaki* is very weak (Figures 3A,B). In contrast, MaGal1 strongly recognizes *P. chesapeaki* (Figure 3C), suggesting that host preference of the parasite for either bivalve host may be related to galectin-mediated recognition and entry into the hemocyte.

Based on these results we characterized the oyster and clam galectins in their molecular, structural, and carbohydrate-binding properties, and proceeded to identify and characterize their carbohydrate ligands on the surface of their hemocytes and the *Perkinsus* trophozoites. Accomplishment of these goals would contribute further understanding of the potential roles of bivalve galectins as soluble and hemocyte-associated receptors for *Perkinsus* parasites. These studies will be discussed in the following sections, and mostly illustrated with results obtained on CvGal1 (Tasumi and Vasta, 2007; Feng et al., 2013; Kurz et al., 2013).

## Biochemical Characterization of Oyster and Clam Galectins

**Figure 4:**
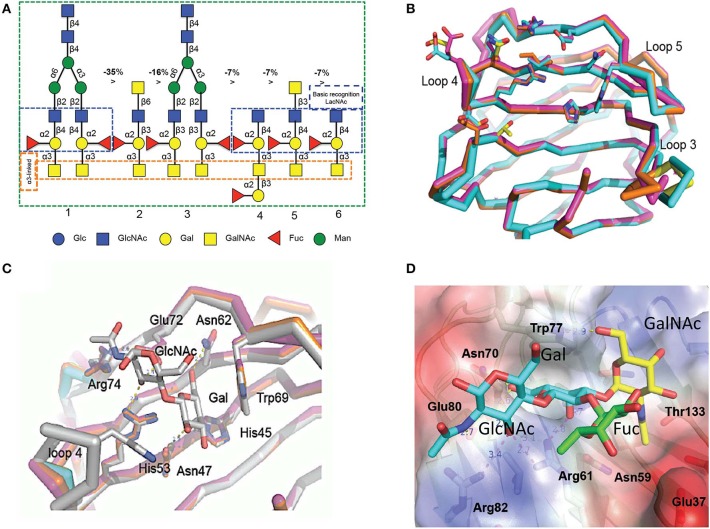
Binding ligand analysis through glycan array and homology modeling. **(A)** Six best glycans ranked by their affinity for CvGal1 in glycan array analysis. The negative percentages are the evaluation of the % change in the fluorescent signal (*F*_*j*_ – *F*_*i*_)/*F*_*i*_ × 100%). **(B)** Model of the four CRDs of CvGal1: Chain A in orange, chain B in cyan, chain C in magenta and chain D in yellow. **(C)** Overlay between the modeled CvGal1 CRDs and BaGal1: Loop 4 of the CvGal1 CRDs (chain colors as in A above) is shorter than the loop 4 of BaGal1 (in gray), allowing bulkier structure next to the N-acetylglucosamine residue. **(D)** A2 blood oligosaccharide docked at the binding pocket of the CvGal1 model of the first CRD, using the observed common *N*-acetyllactosamine disaccharide bound to the template. CvGal1-binding site is shown as semi-transparent solvent-accessible surface colored by its vacuum electrostatic potential (positive in *blue* to negative in *red*). The schematic model of the protein is visible across the surface showing the interacting residues in a *stick* representation. H-bonds recognizing hydroxyl groups of the A2 oligosaccharide are displayed as *dashed lines* with their distances (in Å) between heavy atoms indicated [Adapted from Feng et al. (2013) with permission from the American Society for Biochemistry and Molecular Biology].

The initial characterization of the carbohydrate specificity of CvGal1 using a panel of mono-, oligo-, and polysaccharides, and glycoproteins revealed that CvGal1 has a preference for GalNAc relative to Gal or LacNAc, which are typically recognized by most galectins (Tasumi and Vasta, 2007). Furthermore, the glycoproteins porcine stomach mucin (PSM), asialofetuin, thyroglobulin, lactoferrin, and laminin behaved as strong inhibitors, whereby PSM is a complex mixture of glycans, rich in blood group ABH moieties. To elucidate the fine specificity of CvGal1, the binding of recombinant CvGal1 to a glycan microarray was analyzed at the Core H, Consortium for Functional Glycomics, Emory University, Atlanta. The study confirmed the preliminary results by revealing that CvGal1 preferentially binds to carbohydrates containing non-reducing terminal GalNAc (Tasumi and Vasta, 2007). The strongest binders in the glycan microarray were complex bi-antennary oligosaccharides carrying blood group A type 2 moieties, followed by the type 1 structures and, with less affinity, similar type-2 B oligosaccharides (Figure 4A; Feng et al., 2013).

In contrast with rCvGal1, the glycan array binding profile of rCvGal2 showed that it recognizes with high affinity carbohydrates displaying either blood group A or B type 2 moieties (Feng et al., 2015). Thus, like CvGal1, CvGal2 also prefers type-2 backbone structures Galβ1-4GlcNAc in LacNAc and Gal, and a Fuc linked in α1-2 to the subterminal Gal. Unlike most galectins described to date, which recognize oligosaccharides exhibiting galactosyl units at the non-reducing end, such as *N*-acetyllactosamine moieties (Leffler et al., 2004; Vasta and Ahmed, 2008), our studies revealed that the oyster galectins CvGal1 and CvGal2 preferentially bind to ABH blood groups (Tasumi and Vasta, 2007; Feng et al., 2013, 2015). Unlike CvGal1 and CvGal2, however, a glycan microarray analysis of MaGal1, confirmed the preliminary solid phase assays that suggested preference for galactosyl moieties, and showed that MaGal1 preferentially recognizes Galα1-3Galβ1-4GlcNAcβ1-2Manα1-6(Galα1-3Galβ1-4GlcNAcβ1-2Manα1-3)Manβ1-4GlcNAcβ1-4GlcNAcβ-R and other bi-antennary structures with non-reducing terminal Galα1-3Galβ1-4GlcNAc, followed by those with terminal Galα/β1-4Galβ1-4GlcNAc (Vasta et al., 2015).

To elucidate the structural basis for the binding specificity of CvGal1 and CvGal2 we modeled their structure using the toad *Bufo arenarum* galectin-1 (BaGal1) as template (Bianchet et al., 2000) and analyzed the interactions of the CRDs with the preferred carbohydrate ligands for both oyster galectins identified in the solid phase assays and glycan array analysis (Figures 4B–D). The structures of the four CRDs of CvGal1 overlap very closely with each other (Figure 4B) and with BaGal1 (Figure 4C). Only minor differences among the four CRDs, mostly located in loops 3, 4, and 5, were predicted by the CvGal1 model (Figure 4B). Alignment of the CvGal1 CRDs with BaGal1 showed that amino acid residues that are involved participate in the recognition of the Galβ(1-4)GlcNAc by BaGal1 are mostly conserved in all four CvGal1 CRDs (Feng et al., 2013). In BaGal1 these residues establish interactions with the galactoside moieties as follows (Figures 4C,D): [1] (Arg^49^, His^45^, Asn^47^)−4-OH of Gal, [2](Arg^49^, Glu^72^)—[3-OH in core 1, or 4-OH in core 2 galactosides] of GlcNAc, [3](Asn^62^, Glu^72^)-−5-OH of Gal, and [4] Trp^69^–ring of Gal. The modeling alignment also revealed differences in the secondary structure elements of CvGal1's CRDs with respect to the BaGal1 template, such as the short loop 4 (the sequence between strands 4 and 5) (Figure 4C), as discussed above (Figures 2A,B). In BaGal1 and other vertebrate proto-type galectins only loop 4 participates in the recognition of the galactose moiety, whereby a histidine residue (His^53^) makes an apolar contact with the C2 and O2 atoms of the Gal moiety of the core 2 galactoside. This histidine residue is absent in the short loop 4 of all CvGal1 CRDs (Feng et al., 2013).

To rationalize the recognition of ABH blood group oligosaccharides by CvGal1, the type 2 blood group A oligosaccharide (A2) was docked into the first CvGal1 CRD as guided by the *N*-acetyllactosamine bound to BaGal1 (Feng et al., 2013) (Figure 4D). The space generated by the shortening of loop 4 enabled fitting the 2'-fucosyl moiety common to both the A and B blood group tetrasaccharides, with its 6-methyl group placed on top of the arginine that coordinates the axial 4- and equatorial 3-OH groups of the first and second moieties of the core galactoside, respectively (Figure 4D). Thus, the glutamate at the tip of loop 4 holds this conserved arginine in position. Distances from Glu^37^, in the 1st CRD, Asn^199^ in the 2nd CRD; and Glu^449^ and Glu^474^, in the 4th CRD to the fucose 4-OH group could support water-mediated bridges, although no direct interaction of polar group from the protein with any fucose hydroxyls could be established (Feng et al., 2013). Similarly, the CvGal2 structural model enabled a visual interpretation of the glycan array results, in particular the preferential binding to ABH blood group bi-antennary structures, and the recognition of sulfated moieties and Forssman antigens (Feng et al., 2015). In contrast with CvGal1, the glycan array analysis revealed that CvGal2 recognizes oligosaccharides displaying both blood group A and B moieties. Like in CvGal1 (Feng et al., 2013) and CGL2 (Walser et al., 2004), the longer loop 3 and shorter loop 4 in CvGal2 as compared to the typical proto type galectins (Bianchet et al., 2000; Feng et al., 2013) supports the structural basis for a preference for blood group oligosaccharides. In blood group A, the methyl-group of the *N*-acetyl moiety in the non-reducing GalNAc is coordinated by residues from a pocket formed on the N-terminus, similar to that observed in the CGL2-A2 antigen complex (Walser et al., 2004). As for CvGal1, the shorter loop 4 enables accommodating simultaneously the 2'-fucosyl group and the α(1-3)Gal[NAc] of A and B moieties. This feature results in an apparent higher affinity relative to that of the vertebrate prototype galectins that possess the typical long loop 4 (Liao et al., 1994; Vasta et al., 2015). It is noteworthy that in all four CvGal2 CRDs (A-D, or 1-4) sequence variations at key positions are suggestive of differences in their fine specificity for glycans displaying ABH moieties (Feng et al., 2015).

**Figure 5:**
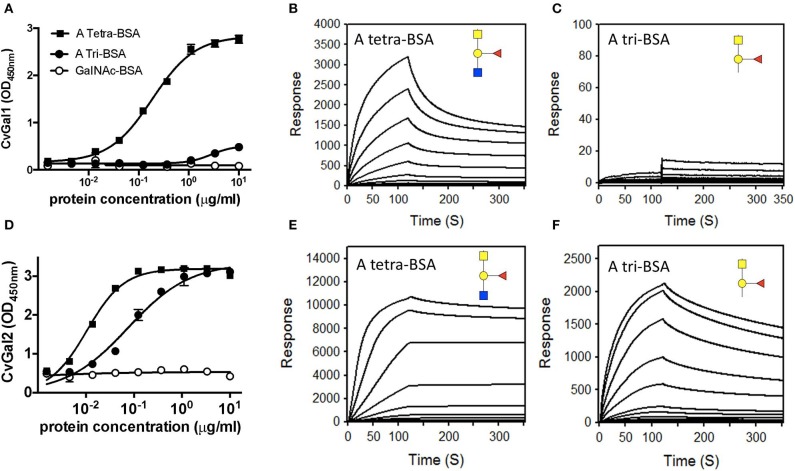
Binding of recombinant CvGal1 and CvGal2 to blood group A antigen. **(A,D)** blood group A tetrasaccharide-BSA (A Tetra-BSA), blood group A trisaccharide-BSA (A Tri-BSA), or *N*-acetylgalactosamine-BSA (GalNAc-BSA) were added at the concentrations indicated (serial dilution starting from 10 μg/ml, 100 μl/well) into 96-well plates and the binding of 0.2 μg/ml of rCvGal1 **(A)** or rCvGal2 **(D)** was assessed by ELISA. Data show optical density at 450 nm (OD_450nm_) in triplicates with standard error (SEM). **(B,C,E,F)** Blood group A tetrasaccharide-BSA (A Tetra-BSA) or blood group A trisaccharide-BSA (A Tri-BSA) were immobilized up to 1,000 response units on CM5 chips, and the binding of rCvGal1 **(B,C)** or rCvGal2 **(E,F)** was assessed by SPR with either lectin being injected as analyte. The SPR sensorgrams were recorded with 2-fold serial dilutions of the analyte starting from 100 μg/ml. Negligible responses were observed on sensorgrams for the GalNAc-BSA (data not shown) [Adapted from Feng et al. (2013) with permission from the American Society for Biochemistry and Molecular Biology].

The experimental validation of the CvGal1 and CvGal2 models, was carried out by first comparatively assessing by ELISA the binding of the recombinant galectins to neoglycoproteins displaying either a monosaccharide (GalNAc), a blood group A trisaccharide [GalNAcα3(Fucα2)Gal], or a type 2 tetrasaccharide [GalNAcα3(Fucα2)Galβ3GlcNAc] conjugated to BSA (Feng et al., 2013). The results showed that CvGal1 strongly bound to the blood group A tetrasaccharide, while the trisaccharide-BSA and GalNAc-BSA were not recognized (Figure 5A). A similar study using an anti-A monoclonal antibody showed that it equally recognized both the blood group A tri- and tetrasaccharides, but displayed negligible binding to GalNAc-BSA. A quantitative analysis of binding affinity by SPR revealed that rCvGal1 binds to the neoglycoprotein with conjugated blood group A type 2 tetrasaccharide with a K_D_ value of 1.5 μM (Figure 5B), while no binding was observed to blood group A trisaccharide-BSA (Figure 5C), or neoglycoproteins displaying GalNAc, Gal or GlcNAc (results not shown) (Feng et al., 2013). A similar analysis showed that in contrast with CvGal1, CvGal2 recognized both the blood group A tetra- and the trisaccharide-BSA (Figure 5D; Feng et al., 2015). SPR analysis confirmed that CvGal2 recognized both blood group A tetra- and trisaccharide-BSA, but displayed higher affinity for the tetrasaccharide than the trisaccharide (4.8 and 60 nM, respectively) (Figures 5E,F). Further SPR assessment of CvGal2 binding affinity with various blood group ABH oligosaccharides showed that it binds to type 1, 2, and 3/4 of blood group A tetrasaccharides (K_D_ = 2.5–21 μM) (Feng et al., 2015). In conclusion, although CvGal1 and CvGal2 are structurally similar, their fine specificities for ABH blood group oligosaccharides are both quali- and quantitatively different. Ongoing studies in our lab are aimed at expressing the recombinant individual CRDs from both CvGal1 and CvGal2 to enable the identification of potential differences in binding specificity among them.

## Identification of Galectin Ligands on the Hemocyte and Parasite Surface

**Figure 6:**
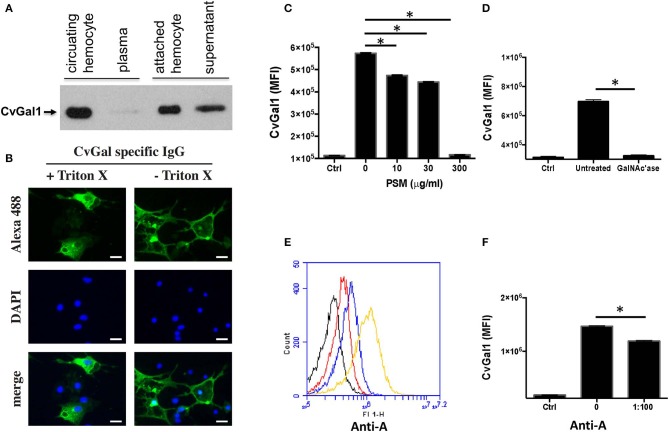
Binding of CvGal1 and CvGal2 to oyster hemocytes. **(A)** Upon hemocyte attachment and spreading, CvGal1 is translocated to the periphery and secreted. Western blotting of unattached hemocytes, plasma, attached-spread hemocytes, and supernatant. **(B)** Immunofluorescence staining with anti-CvGal1 and DAPI staining of attached-spread hemocytes with (+) or without (–) Triton X treatment, showing the presence of CvGal1 in the cytoplasm, and on the external surface of the hemocyte plasma membrane, respectively. Scale bar, 10 μm. **(C)** Binding of rCvGal1 (100 μg/ml) to hemocytes in the presence of PSM (0–300 μg/ml), whereby the control (*Ctrl*) is sample without exogenous rCvGal1 and inhibitor. **(D)** Binding of rCvGal1 to unattached hemocytes with α-*N*-acetylgalactosaminidase treatment (*GalNAc'ase*) or no treatment (*Untreated*) were measured by flow cytometry, whereby a sample without rCvGal1 was the control (*Ctrl*). Data show mean fluorescence intensity (*MFI*) ± S.E. of each sample. **(E)** Fixed hemocytes were stained with dilutions of anti-blood group A antibody (*red*, 1:2000; *blue*, 1:500; *yellow*, 1:100) or buffer only (*black*) in flow cytometry analysis. **(F)** Fixed cells were preincubated with anti-blood group A antibody (1:100), and the binding of rCvGal1 (100 μg/ml) was measured by flow cytofluorometry; the control (*Ctrl*) was recorded in the absence of rCvGal1. *Indicates significant difference (*p* < 0.05) between samples from One-Way ANOVA analysis [Adapted from Tasumi and Vasta (2007) and Feng et al. (2013) with permission from the American Association of Immunologists and the American Society for Biochemistry and Molecular Biology].

As discussed above, analysis of CvGal1 expression suggested that it takes place in the hemocytes (Tasumi and Vasta, 2007; Feng et al., 2013). We experimentally assessed the subcellular localization of the CvGal1 protein, by raising an antiserum against rCvGal1, and validating the specificity of the purified anti-rCvGal1 immunoglobulins by Western blot against a crude hemocyte extract, using rCvGal1 as control (Tasumi and Vasta, 2007). In agreement with the detection of the CvGal1 transcripts in hemocytes, the Western blot results revealed that the mature protein was also localized in the cytoplasm of the circulating hemocytes, suggesting that the gene is transcribed, translated, and the protein accumulates in the hemocyte. Because upon attachment and spreading on a foreign surface, the oyster hemocytes become motile and avidly phagocytic, we examined by blotting the presence of CvGal1 in plasma and hemocytes [circulating (unattached), and attached/spread]. Both the circulating and attached hemocytes showed a strong CvGal1 band, whereas the attached hemocytes secreted soluble CvGal1 into the extracellular space (Figure 6A). Next, we used immunofluorescence to assess the subcellular distribution of CvGal1 in unattached and attached hemocytes (Figure 6B). In the unattached hemocytes, CvGal1 was localized to the cytoplasm of approximately one third of the permeabilized hemocytes (+ Triton-X), but no signal was observed in the untreated cells (– Triton-X). In contrast, in both permeabilized and untreated attached hemocytes, intense diffuse staining was observed approximately in the same proportion as in the unattached permeabilized cells (Tasumi and Vasta, 2007). Based on their subcellular morphology under phase contrast microscopy, these cells were identified as granulocytes (Kennedy et al., 1996; Terahara et al., 2006). Unlike the CvGal1 localization in circulating hemocytes, the high concentration of CvGal1 detected on the plasma membrane of the untreated attached hemocytes, particularly on the surface of filopodia (Figure 6B), suggest that upon attachment and spreading, the cytoplasmic CvGal1 is secreted to the extracellular space and binds to the hemocyte surface glycans. Galectins are secreted by an unconventional mechanism not yet fully elucidated, as they lack the signal peptide typical of secreted proteins (Hughes, 1999). Therefore, galectins are not exported via the typical secretory pathway but through a mechanism that resembles vesicle exocytosis (Hughes, 1999). CvGal1, which also lacks a signal peptide, may be no exception to this observation, and may be secreted by the non-classical mechanism common to other galectins (Tasumi and Vasta, 2007). We also examined the possibility that the CvGal1 released by the attached granulocytes binds to the external surface of circulating hemocytes by binding to surface moieties. For this, we tested by immunofluorescence the potential binding of rCvGal1 to unattached hemocytes (Tasumi and Vasta, 2007). Intense staining was observed in virtually 100% of the cells examined, indicating that CvGal1 can strongly bind to both attached and unattached hemocytes subpopulations, including hyalinocytes. These observations suggest that the CvGal1 secreted by the attached granulocytes binds to carbohydrate moieties on the cell surface and upon saturation, the secreted galectin remains in plasma as a soluble protein (Tasumi and Vasta, 2007). Similar observations were made with CvGal2 (Feng et al., 2015).

Based on our studies on the specificity of CvGal1 and CvGal2 for ABH blood group moieties described above, we addressed the question about the identity of the carbohydrate moieties on the hemocyte surface that are recognized by the oyster galectins (Feng et al., 2013, 2015). For this, we used flow cytometry, to assess the binding of CvGal1 to fixed, non-permeabilized hemocytes and its inhibition by increasing concentrations of PSM, a mixture of glycoproteins rich in ABH moieties, using BSA as control (Feng et al., 2013). Strong binding of CvGal1 to the hemocyte surface was observed, and this interaction could be specifically prevented by pre-incubation of CvGal1 with PSM (300 μg/ml) (Figure 6C). Pre-treatment of the hemocytes with α-*N*-acetylgalactosaminidase also significantly prevented CvGal1 binding (Figure 6D), confirming that non-reducing terminal αGalNAc on the hemocyte surface is required for CvGal1 binding. Monoclonal anti-A antibodies strongly bound to the hemocyte surface (Figure 6E) revealing that exposed blood group A moieties are present, and that the αGalNAc detected may possibly be the terminal moiety on the hemocyte ligands recognized by CvGal1 (Tasumi and Vasta, 2007). In contrast, the anti-B antibodies failed to bind to the intact hemocytes. Furthermore, we observed partial inhibition of rCvGal1 binding to the intact hemocytes by anti-A monoclonal antibodies, and *vice versa*, that pre-treatment with anti-A antibodies could partially inhibit binding of CvGal1(Figure 6F), whereas the anti-B antibodies had no effect (Feng et al., 2013). Taken together, these observations suggested that blood group A moieties on the hemocyte surface are, at least in part, the ligands for CvGal1.

**Figure 7:**
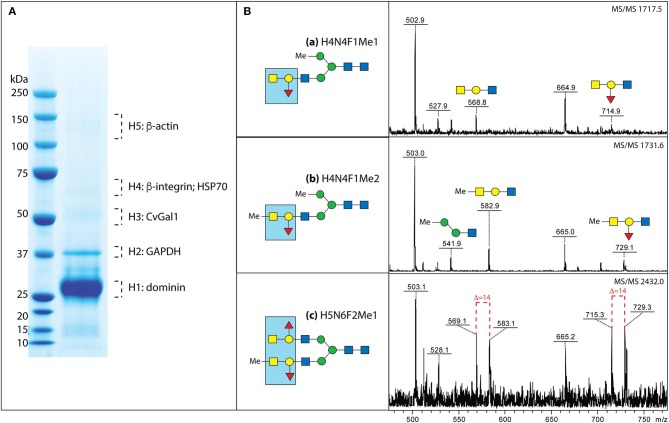
Binding ligands of CvGals on oyster hemocyte surface. **(A)** Isolation and identification of CvGal1 ligands from oyster hemocytes. Oyster hemocyte extracts were eluted from a rCvGal1-column (rCvGal1 cross-linked to Affi-Gel 15, BioRad) using 50 mM lactose, prior to analysis by SDS-PAGE. The Coomassie blue-stained protein bands (H1–H5) were excised from the gel and subjected to proteomic analysis as described (Feng et al., 2013). Peptide sequences were identified using Mascot software to search the NCBInr 167 database, processed in Scaffold and the proteins identified based on the identification of at least two peptides at 95% or greater confidence. **(B)** MS/MS analysis of oyster glycans carrying the histo-blood group A modification. *a–c, N*-glycans from selected NP-HPLC fractions (6.0, 5.7, and 7.7 g.u., respectively) were subject to MS/MS of their protonated forms in positive mode with the focus on the fragments in the range *m/z* 500–800, which are indicative of modification by the blood group A and methyl groups; for the glycan carrying both non-methylated and methylated forms of the blood group A, the differences of 14 Da between the sets of fragments are shown with *dashed lines*. The blue boxes emphasize the identified blood group A tetrasaccharide structure (for further details, see Feng et al., 2013 and Kurz et al., 2013).

Next, we used affinity chromatography of the oyster hemocyte extracts on a rCvGal1-Affi-Gel 15 column to isolate the CvGal1 ligands, and selected bands from SDS-PAGE were subjected to proteomic analysis for the identification of these glycans (Feng et al., 2013). The mass spectrometry analysis identified multiple peptides that matched β-integrin, dominin, GAPDH, and HSP70 in the bands of the expected electrophoretic mobilities (Figure 7A; Feng et al., 2013). Dominin is a major plasma protein that houses a Cu/Zn superoxide-like domain, and is highly similar to cavortin, an iron binding-protein from the Pacific oyster (*C. gigas*) (Itoh et al., 2011). Iron is critical for *P. marinus* intracellular survival in oyster hemocytes (Schott and Vasta, 2003; Schott et al., 2003a; Fernández-Robledo et al., 2008; Alavi et al., 2009), and the iron transporters (Nramp; Robledo et al., 2004; Lin et al., 2011) in both the parasite and the oyster host are involved in their competition for available iron (Cellier et al., 2007). Another interesting ligand for CvGal1 identified on the hemocyte surface is β-integrin (Zhuo et al., 2008; Feng et al., 2013), and this was of particular interest as this transmembrane signaling glycoprotein is a key receptor in cell activation processes (Mayadas and Cullere, 2005; Lim and Hotchin, 2012). We have observed that the addition of PSM to attached and spread hemocytes, promotes activation and phagocytosis of *P. marinus*. It is possible that this results from clustering of hemocyte β-integrin-bound CvGal1 by the multivalent PSM, which acts as a three-component glycoprotein-CvGal1-β-integrin lattice that leads to cell activation.

A rigorous *N*-glycomic study on selected glycoproteins isolated on a CvGal1 column and identified by proteomic analysis, together with plasma and hemocyte glycoproteins, demonstrated the presence of blood group A oligosaccharide moieties on some hemocyte N-glycans (Kurz et al., 2013; Figure 7B). Frequent methylation and sulfation of the identified hemocyte glycans was observed, with the latter likely to confer a significant negative charge to the hemocyte glycocalyx (Kurz et al., 2013). These observations are consistent with the results of our CvGal1-ligand interaction model which revealed that methyl groups can be present at the 4-OH and 6-OH of the GalNAcα(1-3), without any negative effect on CvGal1 recognition by the protein (Feng et al., 2013). The results of the glycomic analysis are supported by identification of an α1,2-fucosyltransferase gene in the oyster genome, which is predicted to encode an enzyme that can transfer L-Fuc to Gal (Zhang et al., 2012).

**Figure 8:**
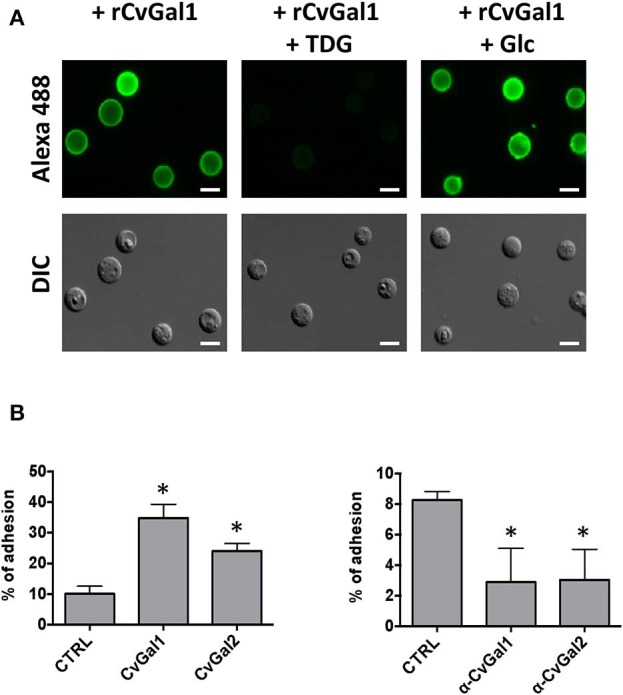
CvGals mediate *P. marinus* trophozoites adhesion onto oyster hemocytes. **(A)** Carbohydrate -specific binding of rCvGal1 to *P. marinus* trophozoites in the presence and absence of thiodigalactose (inhibits rCvGal1 binding) or glucose (does not inhibit rCvGal1 binding) was analyzed by immunofluorescence staining. Scale bar, 10 μm. **(B)** Adhesion of *P. marinus* trophozoites to hemocytes was enhanced by addition of recombinant CvGal1 or CvGal2 (left) or inhibited by addition of anti-CvGal1 or anti-CvGal2 antibody (right). *Indicates significant difference (*p* < 0.05) from control sample [Adapted from Tasumi and Vasta (2007), and Feng et al. (2013) with permission from the American Association of Immunologists and the American Society for Biochemistry and Molecular Biology].

**Figure 9:**
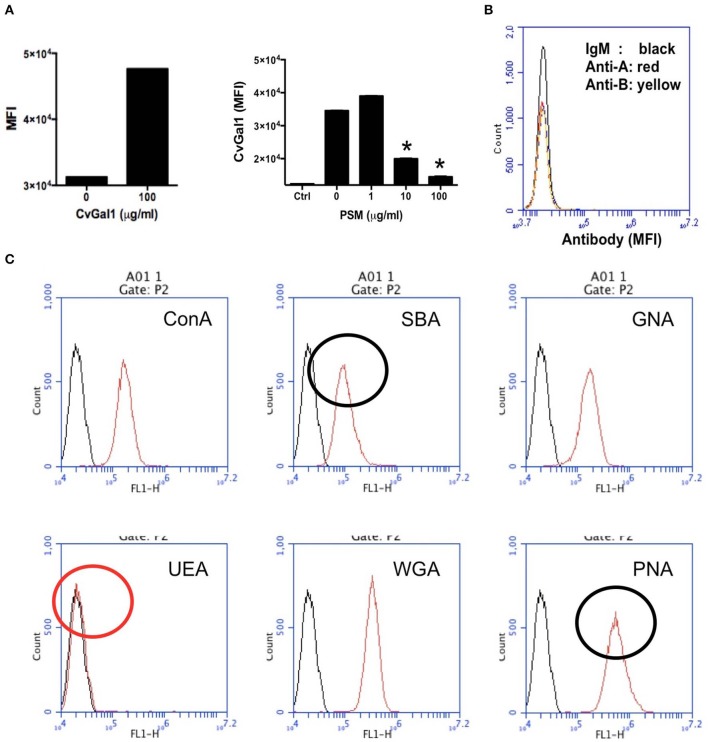
Binding of rCvGal1 to *P. marinus* trophozoites. **(A)** Binding of rCvGal1 (100 μg/ml) to *P. marinus* trophozoites was measured by flow cytometry analysis. Data show mean fluorescence intensity (*MFI*) ± S.E. of each sample. The binding of rCvGal1 (100 μg/ml) to *P. marinus* trophozoites in the presence of PSM (0–100 μg/ml) is shown on the right hand panel of A. *Indicates significant difference (*p* < 0.05) from sample without PSM inhibition (*0*). Sample without exogenous rCvGal1 and inhibitor was shown (*Ctrl*). **(B)** anti-A or anti-B binding to *P. marinus* revealed the absence of exposed A and B blood group moieties. **(C)** *P. marinus* trophozoites were stained with fluorochrome-labeled lectins (*red lines*) or buffer alone (*black lines*) in flow cytometry analysis. Black circles indicate significant staining of SBA (soybean agglutinin) and PNA (peanut agglutinin) over background, and red circle indicates no significant staining of UEA (*Ulex europaeus* agglutinin) [Adapted from Feng et al. (2013) and Kurz et al. (2013) with permission from the American Society for Biochemistry and Molecular Biology].

We then carried out experiments to identify the nature of the glycan moieties recognized by CvGal1 and CvGal2 on the surface of the *Perkinsus* trophozoites. CvGal1 binds strongly and in a carbohydrate-specific manner to *P. marinus* trophozoites (Figure 8A; Tasumi and Vasta, 2007). The binding can be fully prevented by thiodigalactose (TDG), an effective galectin inhibitor, but not by glucose. Furthermore, pre-incubation of *P. marinus* trophozoites with either CvGal1 or CvGal2 enhances their adhesion to the hemocyte surface, whereas pre-treating the hemocytes with anti-CvGal1 or -CvGal2 antibodies significantly decreases adhesion, suggesting that both galectins can function either as opsonins or cell surface receptors for the parasite (Figure 8B; Feng et al., 2013). Based on the identification of blood group A oligosaccharides as the hemocyte surface ligands for the oyster galectins, we investigated the possibility that the CvGal1 ligands on the parasite surface were also ABH blood group moieties. Thus, we first assessed the binding of CvGal1 and its inhibition by PSM by flow cytometry. CvGal1 bound strongly to the trophozoites, and pre-incubation with PSM reduces the binding in a dose-dependent manner, similar to our observations with hemocytes (Figure 9A; Feng et al., 2013). Strikingly, however, we observed no binding of the anti-A or anti-B monoclonal antibodies to the *P. marinus* trophozoites (Figure 9B). This revealed that no blood group A or B moieties are exposed on the parasite surface, and suggested that the GalNAc or related sugars recognized by CvGal1 are linked to different glycan structures (Feng et al., 2013).

To tentatively identify these carbohydrate moieties on the surface of *P. marinus* trophozoites we used labeled plant lectins to carry out a glycotyping analysis of the parasite surface (Figure 9C; Feng et al., 2013). We observed strong signals with ConA (concanavalin A; binding αMan, GlcNAc, and αGlc), SBA (α,βGalNAc and α,βGal), GNA (*Galanthus nivalis* agglutinin; α1-3 and α1-6 high mannose oligosaccharides), WGA (Wheat germ agglutinin; Neu5Ac or β4-linked terminal HexNAc), and PNA (Galβ1-3GalNAcα). UEA (*Ulex europaeus* agglutinin), showed no binding, indicating the absence of exposed Fucα1-2Gal moieties. This was supported by the absence of fucosyltransferase genes in the *P. marinus* genome (Caler et al., in preparation). In contrast, the binding of SBA and PNA to *P. marinus* trophozoites revealed the presence of exposed GalNAc and Gal moieties on the parasite surface. The absence of Fucα1-2Gal and lack of anti-A and anti-B antibody staining suggest that the strong binding of CvGal1 and CvGal2 to the parasite surface is based on the recognition of exposed GalNAc and Gal as components of carbohydrate moieties that may be topologically similar to A or B blood group oligosaccharides, but chemically different (Feng et al., 2013). Ongoing glycomic studies are aimed at the identification and structural characterization of the glycans that function as CvGal1, CvGal2 and MaGal1 ligands on *Perkinsus* trophozoites.

## Summary and Conclusions

Among the various lectin families, galectins are evolutionarily conserved and taxonomically widely distributed lectins endowed with key regulatory and effector functions in multiple biological processes (Leffler et al., 2004; Vasta et al., 2007, 2012; Vasta and Ahmed, 2008; Vasta, 2009; Rabinovich and Croci, 2012)^1^. Within the marine environment, galectins seem to be ubiquitous among both invertebrates (reviewed in Vasta et al., 2004a, 2015; Vasta and Ahmed, 2008; Vasta, 2009; Wang and Wang, 2013) and vertebrates (reviewed in Vasta et al., 2004b, 2011; Shirai et al., 2006; Cummings et al., 2017). By the use of biochemical, molecular, glycomic, and structural approaches we identified and characterized the specificity of the oyster galectins CvGal1, CvGal2 and the clam galectin MaGal1, and have gained novel insights into the nature of their carbohydrate ligands on the surfaces of the oyster hemocyte and *Perkinsus* parasites (Tasumi and Vasta, 2007; Feng et al., 2013, 2015; Vasta et al., 2015). This work is ongoing in our lab and those of our collaborators, and we aim to achieve a detailed and comprehensive view of the protein-carbohydrate interactions and mechanisms that determine host preference, recognition, and entry of *Perkinsus* parasites into their bivalve hosts. We expect that the bivalve-*Perkinsus* system will constitute a useful model to address the role(s) of galectins in host defense against parasites, as well as their unique glycan adaptations for host colonization.

The binding profiles of the oyster galectins CvGal1 and CvGal2 in the glycan microarray revealed that they preferentially recognize ABH blood group oligosaccharides, whereas the clam galectin MaGal1 binds to oligosaccharides with terminal Gal. The modeling of the galectins's CRDs and docking of selected ABH oligosaccharides into their binding pockets, enabled a detailed visualization of the interactions that take place between the protein and the carbohydrate ligand. Most galectins typically recognize oligosaccharides exhibiting non-reducing terminal galactosyl moieties, particularly *N*-acetyllactosamine units (Di Lella et al., 2011). Selected galectins, however, may recognize non-reducing terminal GalNAc on surface carbohydrate moieties of parasites or microbial pathogens, such as the human galectin-3 that binds to glycans displaying GalNAcβ1–4GlcNAc from the parasite helminth *Schistosoma mansoni* (van den Berg et al., 2004). Other galectins described in taxa from fungi to mammals display specificity for ABH blood groups (Stowell et al., 2008a,b, 2010). For example, CGL2, a galectin from the mushroom *Coprinus cinereus*, can bind blood group A oligosaccharides (Cooper et al., 1997; Walser et al., 2004), while the mammalian galectins 2, 3, 4, and 8 can recognize both A and B oligosaccharides (Stowell et al., 2008a,b). The glycomic study carried out on selected hemocyte surface glycans recognized by CvGal1 rigorously demonstrated the presence of blood group A oligosaccharides (Kurz et al., 2013), thereby confirming results from biochemical approaches (Tasumi and Vasta, 2007; Feng et al., 2013).

**Figure 10:**
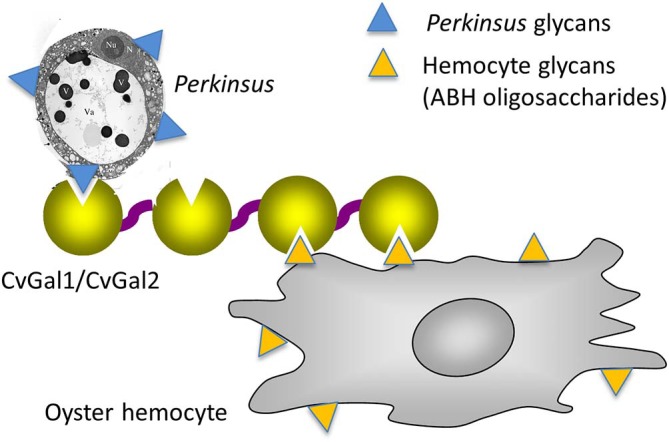
Schematic model of CvGals-mediated *Perkinsus* sp. infection. Yellow triangles represent the binding ligands on hemocytes (mainly ABH oligosaccharides on dominin) and blue triangles represent the chemically different binding ligands on *Perkinsus* sp. parasites.

As mentioned above, the oyster galectins CvGal1 and CvGal2 that have been secreted by the hemocytes upon recognition of a foreign surface or particle, can bind to the hemocyte surface, with some CvGal1 and CvGal2 remaining as soluble protein in extracellular space (Tasumi and Vasta, 2007). The strong recognition of *P. marinus* trophozoites, their enhanced adhesion to hemocytes by pre-incubation with CvGal1 and CvGal2, and the specific inhibition of adhesion and phagocytosis by anti-CvGal1 and anti-CvGal2 antibodies strongly suggest that host galectins can recognize and mediate uptake of phytoplankton, bacteria, and *Perkinsus* parasites either as cell surface receptors or as opsonins by cross-linking glycans on the parasite to those on the hemocyte (Tasumi and Vasta, 2007; Vasta, 2009; Figure 10). The internalized *Perkinsus* parasites use their powerful anti-oxidative machinery to inhibit the hemocyte's typical oxidative burst (Wright et al., 2002; Ahmed et al., 2003; Schott and Vasta, 2003; Schott et al., 2003a,b, 2019; Asojo et al., 2006), they survive and proliferate, and eventually lyse the infected hemocytes (Alavi et al., 2009). The parasite progeny released by the desintegrating hemocytes are phagocytosed by other attached hemocytes, or circulating cells that are activated by the binding of secreted CvGal1 or CvGal2 to their surface and recognition of the released parasites (Tasumi and Vasta, 2007; Feng et al., 2013, 2015; Vasta et al., 2015).

**Figure 11:**
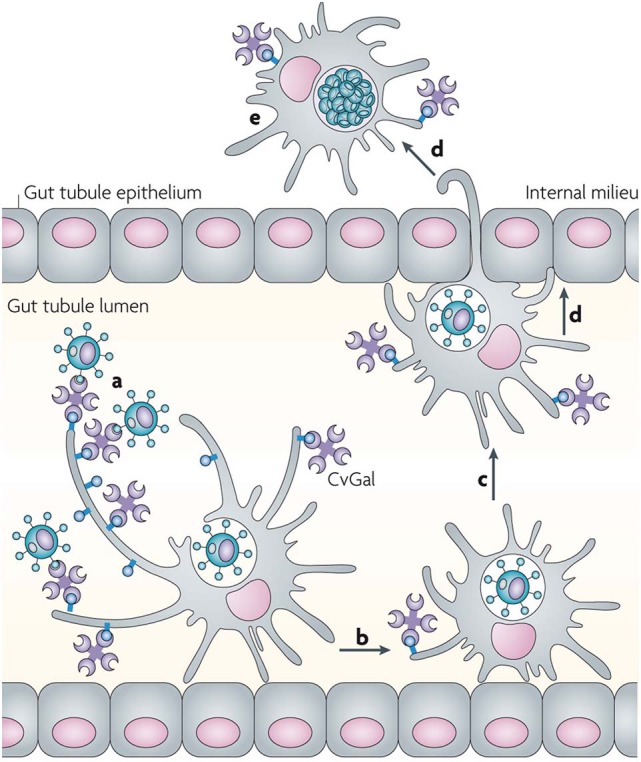
Recognition of *Perkinsus marinus* trophozoites by the oyster (*Crassostrea virginica*) galectin CvGals facilitates infection: CvGals displays four canonical galectin carbohydrate-recognition domains (CRDs), a domain organization that is unlike any of the known galectin types. CvGal is translocated to the periphery and secreted by attached hemocytes, and binds to the cell surface. *P. marinus* trophozoites (a) ingested by filter-feeding are recognized by CvGal on the surface of hemocytes that coat the gut tubules, phagocytosed (b) and transported through the gut epithelium (c,d) into the internal milieu. The parasite inhibits intracellular killing by the host hemocytes and proliferates (e), thereby causing systemic infection and eventually death of the host [Adapted from Vasta (2009) with permission from the Springer Nature].

Although CvGal1 or CvGal2 strongly recognize ABH blood group oligosaccharides on the surface of hemocyte surface, the carbohydrate moieties recognized on the *Perkinsus* surface may be topologically similar, albeit chemically different, as no A or B oligosaccharides could be detected by the specific monoclonal antibodies (Tasumi and Vasta, 2007; Feng et al., 2013, 2015; Kurz et al., 2013). Further, as the presence of exposed GalNAc and Gal moieties on the parasite surface was revealed by glycotyping analysis with labeled plant lectins, the lack of binding of the lectin UEA to the trophozoite surface, suggested that Fuc(α1–2)Gal moieties are absent (Figure 9). Additionally, the absence of fucosyltransferase genes in the *Perkinsus* genome support the lack of *bona fide* A or B oligosaccharides on the trophozoite surface. Based on this experimental evidence it is tempting to speculate that the *Perkinsus* parasite has co-evolved with the oyster host and adapted its glycocalyx to acquire effective mimicry of the “self” ligands recognized by CvGal1 on the hemocyte surface, and gained galectin-mediated entry into the oyster phagocytic hemocytes, survive the oxidative attack, and proliferate (Vasta, 2009; Figures 10, 11). ABH blood group oligosaccharides have been identified in most vertebrate species (Marionneau et al., 2001), as well as in some invertebrates and bacteria (Tasumi and Vasta, 2007; Stowell et al., 2010; Feng et al., 2013, 2015; Kurz et al., 2013). Their taxonomic distribution, however, has not been investigated in a comprehensive manner, and their biological role(s) and evolutionary aspects are subject of current discussion (Marionneau et al., 2001; Gagneux et al., 2017; Stanley and Cummings, 2017).

In recent years, evidence has accumulated to support a role of galectins as carbohydrate-specific receptors that recognize surface carbohydrate moieties of viruses, bacteria and parasites, although in very few examples the identity of the recognized moieties has been rigorously identified (Vasta, 2009). Based on these observations, galectins are often considered to function as PRRs. However, the Janeway and Medzhitov model (Janeway and Medzhitov, 2002) for self/non-self recognition, proposes that PRRs recognize pathogen- or microbe-associated molecular patterns (PAMPs or MAMPs), such as LPS or peptidoglycan, that are highly conserved and widely distributed among viruses, microbes, and parasites, but are absent from the host. Therefore, galectins do not fit a *sensu stricto* definition of PRRs as they can recognize endogenous (“self”) and exogenous (“non-self”) carbohydrate moieties with the same binding site. This apparent paradox reveals significant gaps in our understanding of galectin-carbohydrate binding equilibrium dynamics, the subcellular compartmentalization and secretion of galectins and their carbohydrate ligands, as well as structural and biophysical aspects of their recognition of multivalent glycans (Dam and Brewer, 2008; Vasta, 2009; Vasta et al., 2012). Although substantial progress has been achieved in the past few years, these aspects warrant further investigation.

## Author Contributions

GV wrote the article draft and all co-authors listed have made a substantial, direct and intellectual contribution to the work, and approved it for publication.

### Conflict of Interest

The authors declare that the research was conducted in the absence of any commercial or financial relationships that could be construed as a potential conflict of interest.

## Footnotes

^1^A genomics resource for animal lectins. Available online at: http://www.imperial.ac.uk/research/animallectins/

## Abbreviations

CvGal1: *Crassostrea virginica* galectin 1
CvGal2: *Crassostrea virginica* galectin 2
MaGal1: *Mya arenaria* galectin 1
BaGal1: *Bufo arenarum* galectin-1
hGal-1: human galectin-1
CGL2: *Coprinus cinereus* galectin-2
CRD: carbohydrate recognition domain
GalNAc: N-acetyl-D-galactosamine
GlcNAc: N-acetyl-D-glucosamine
LacNAc: N-acetyllactosamine
TDG: thiodigalactose
BSM: bovine submaxillary mucin
OSM: ovine submaxillary mucin
PSM: porcine stomach mucin
SPR: surface plasmon resonance
PRR: pattern recognition receptors.
